# Psychological symptoms and loneliness in unemployed people diagnosed with mental illnesses

**DOI:** 10.1007/s00127-024-02806-y

**Published:** 2024-12-27

**Authors:** Felix S. Hussenoeder, Maria Koschig, Ines Conrad, Alexander Pabst, Katharina Gatzsche, Luise Bieler, Mathias Alberti, Katarina Stengler, Steffi G. Riedel-Heller

**Affiliations:** 1 Institute of Social Medicine, Occupational Health and Public Health, Leipzig University, Ph.- Rosenthal-Str. 55, 04103 Leipzig, Germany; 2 Helios Park-Klinikum - Clinic for Psychiatry, Psychosomatics and Psychotherapy, Leipzig, Germany

**Keywords:** Psychological symptoms, Loneliness, Unemployment, Mental illness, SCL-90

## Abstract

**Purpose:**

Loneliness is a pervasive phenomenon that is linked to adverse health outcomes. Unemployed individuals with mental illnesses (UMIs) constitute a high-risk group, with substantial implications for both health and vocational (re)integration. This study aims to gain deeper insights into the relationships between psychological problems and symptoms of psychopathology and loneliness in UMIs.

**Methods:**

Our research is based on a sample from LIPSY, a project that aims to maintain or restore employability. Two regression analyses were conducted on a sample of unemployed participants diagnosed with a mental illness (ICD-10: F-code) with the outcome variable loneliness (UCLA). In the first analysis, age, gender, education, cohabitation status, and social network size (LSNS-6) were used as predictors; in the second one, the nine symptom dimensions (SCL-90) - (1) Somatization, (2) Obsessive-Compulsive, (3) Interpersonal Sensitivity, (4) Depression, (5) Anxiety, (6) Anger-Hostility, (7) Phobic Anxiety, (8) Paranoid Ideation, (9) Psychoticism -were added.

**Results:**

Our sample included 397 participants with an average age of 35.8 years, 53.1% were female. The final regression showed significant positive associations between higher levels of education, Interpersonal Sensitivity, Depression, and the outcome loneliness, and a significant negative relationship between Somatization and loneliness.

**Conclusion:**

The high scores on all SCL-90 dimensions, and the links identified between Somatization, Interpersonal Sensitivity, Depression, and loneliness highlight the importance of psychological screening and/or diagnostics in this high-risk group and offer several starting points for prevention measures as well as interventions.

## Introduction

Loneliness is a pervasive phenomenon [1], and is often linked to impaired health outcomes, from cognitive decline in older adults [2], to depression [3], substance abuse [4], eating disorders [5], and cancer [6] The potential causes of loneliness discussed in the literature are numerous and diverse, including shyness [7], stress [8], and problematic internet use [9]. In addition, multiple risk groups were identified by researchers, including ethnic minorities, single-person households, young women, adolescents with a low level of education, and unemployed as well as mentally ill individuals [10]. It is reasonable to conclude that different (combinations of) factors are associated with the development and maintenance of loneliness in different risk groups. To better identify care needs and develop effective prevention interventions, it is therefore important to focus on and target specific risk groups.

In this study, we concentrate on a high-risk group: unemployed individuals diagnosed with a mental illness (UMIs). Not only do they combine two major risk factors for loneliness, but research also shows that unemployment and mental illness can reinforce each other, creating a so-called “vicious circle” [11–13]. The underlying mechanisms may include, for example, the loss of economic resources and social relationships due to unemployment, as well as the stigmatization of people with mental illnesses in the labor market. Moreover, research indicates that there are detrimental interactions between loneliness and unemployment [14] as well as between loneliness and mental health [15, 16], which could contribute to further stabilization and reinforcement, making loneliness a key factor in health and occupational (re)integration.

The considerable complexity of mental illnesses, with notable inter-individual variability, presents a significant challenge in identifying the specific symptoms within these syndromes that are responsible for the detrimental relationships between mental health and loneliness. Hence, the aim of our study is to focus on the symptom level and to investigate the relationships between psychological problems and symptoms of psychopathology – i.e., SCL-90 dimensions (1) Somatization, (2) Obsessive-Compulsive, (3) Interpersonal Sensitivity, (4) Depression, (5) Anxiety, (6) Anger-Hostility, (7) Phobic Anxiety, (8) Paranoid Ideation, and (9) Psychoticism [17] - and loneliness, in order to identify those that are most relevant for prevention and therapy. The literature shows positive associations between the average symptom score over all dimensions and loneliness, and between Interpersonal Sensitivity, Depression, and loneliness in a sample of US college students in the early 1990s [18, 19]. In addition, scholars found negative associations between social support and all of the SCL-90 dimensions [20–22]. Our study fills a research gap by providing a detailed analysis of associations between symptom dimensions and loneliness in a highly vulnerable group, i.e., UMIs, and in a European setting. A more profound and empirically-based understanding of those interconnections will contribute to better public mental health and to the alleviation of the burden on the social system.

## Methods

### Study design

The present study is based on a sample from LIPSY, a project that aims to maintain and/or restore the employability of unemployed people with mental illnesses receiving means-tested benefits and enable them to (re)integrate into the labor market via targeted advice and, in some cases, personalized coaching [23]. Potential participants were recruited at a job center in a large German city. If they were screened positive for mental health problems, they were invited to participate in the LIPSY project and the accompanying evaluation.

The evaluation was conducted using self-assessment questionnaires at four points in time, at baseline and after 6, 12, and 18 months, including a comprehensive psychiatric assessment at baseline. The initial 600 questionnaires from the baseline assessment were utilized for the purpose of this study. After removing all cases that did not receive at least one confirmed diagnosis of a mental illness from a professional psychotherapist (ICD code F, *n* = 44) and all questionnaires with incomplete data (*n* = 159), the resulting analysis sample consisted of 397 individuals. Among participants, affective disorders (64.0%), anxiety disorders (29.7%) and substance-related disorders (21.2%) were the most common diagnoses (percentages exceed 100 due to comorbidity). More detailed information on diagnoses and their association with age, gender, and the use of care services can be found elsewhere [24].

### Ethics

The study is in accordance with the Helsinki Declaration of 1975 (revised in 2008) and was approved by the Ethics Committee of the University of Leipzig. Written informed consent was obtained from all participants.

### Measures

*Socio-demographic variables*. The participants provided information regarding their age, gender, level of education, and cohabitation with a partner. Education was classified into four categories: (1) no school-leaving certificate, (2) low (secondary school-leaving certificate), (3) medium (intermediate/10th grade polytechnic secondary school-leaving certificate), and (4) high (advanced technical college entrance qualification, technical secondary school-leaving certificate, general/specialized higher education entrance qualification/Abitur).

*Social network size.* The size of the participants’ social networks was assessed with the assistance of the abbreviated Lubben Social Network Scale (LSNS-6; [25]). The scale encompasses six items, which can be responded to on a six-point scale and pertain to connections with family members, friends, and neighbors. In theory, the values range between 0 and 30, with higher values corresponding to larger networks.

*Symptom checklist.* The SCL-90-S is a self-report measure that assesses the subjectively perceived impairment due to physical and psychological symptoms over the past seven days [17]. The 90 items comprising the nine scales delineate the domains of (1) Somatization, (2) Obsessive-Compulsive, (3) Interpersonal Sensitivity, (4) Depression, (5) Anxiety, (6) Anger-Hostility, (7) Phobic Anxiety, (8) Paranoid Ideation, and (9) Psychoticism. Scales may range from 0 to 4. The Global Severity Index (GSI) represents the average score over all 90 items from the SCL-90-S, and it reflects the overall level of psychological distress. The SCL-90-S is the slightly revised version of the SCL-90-R, the results of both versions are comparable [26].

*Loneliness.* Loneliness was assessed using the German 3-item version of the UCLA Loneliness Scale [27, 28], which employs a rating scale ranging from 1 (“never/rarely”) to 3 (“often”). The resulting loneliness scores thus fall between 3 and 9, with higher values representing higher levels of loneliness.

### Statistical analyses

The statistical analyses were conducted using IBM SPSS (version 27). The initial multiple linear regression analysis was performed with the predictors age, gender, education, cohabitation status, and social network size, i.e., the control variable, and the outcome variable loneliness. In the final analysis, the nine SCL-90 dimensions − (1) Somatization, (2) Obsessive-Compulsive, (3) Interpersonal Sensitivity, (4) Depression, (5) Anxiety, (6) Anger-Hostility, (7) Phobic Anxiety, (8) Paranoid Ideation, (9) Psychoticism – were included as predictors.

## Results

The sample consisted of 397 unemployed individuals diagnosed with a mental illness, with an average age of 35.8 years, 53.1% of participants were female. Table 1 provides an overview of the general characteristics of the sample.

**Table 1:** General characteristics of the study population

	Total Group (*N* = 397)
Age (years)	35.8 (11.2)
Gender (“female”)	211 (53.1%)
Education	
No school leaving certificate	44 (11.1%)
Low (secondary general school)	101 (25.4%)
Medium (intermediate/ polytechnic secondary school)	155 (39.0%)
High (technical secondary school, university entrance qualification)	97 (24.4%)
Living with a partner (“yes”)	68 (17.1%)
Social networks size (LSNS-6; Range: 0–30)	10.1 (5.7)
Symptom Dimensions (SCL-90; Range: 0–4)	
Somatization	1.1 (0.8)
Obsessive-Compulsive	1.6 (0.8)
Interpersonal Sensitivity	1.6 (0.9)
Depression	1.8 (0.8)
Anxiety	1.3 (0.8)
Anger-Hostility	1.2 (0.9)
Phobic Anxiety	1.3 (1.0)
Paranoid Ideation	1.3 (0.8)
Psychoticism	0.9 (0.6)
Global Severity Index^1^ (SCL-90; Range: 0–4)	1.4 (0.6)
Loneliness (UCLA; Range: 3–9)	5.9 (1.9)

Note. Continuous variables are given as mean (standard deviation); categorical variables are displayed as numbers (percentages).

^1^The Global Severity Index reflects the overall level of psychological distress a person is experiencing. It represents the average score over all 90 items from the SCL-90.

All symptom dimensions exhibited significant positive correlations with loneliness (*p ≤ 0.05; **p ≤ 0.01; ***p ≤ 0.001): Somatization (*r* = 0.10*), Obsessive-Compulsive (*r* = 0.25***), Interpersonal Sensitivity (*r* = 0.38***), Depression (*r* = 0.37***), Anxiety (*r* = 0.23***), Anger-Hostility (*r* = 0.19***), Phobic Anxiety (*r* = 0.22***), Paranoid Ideation (*r* = 0.30***), Psychoticism (*r* = 0.30***).

Table 2 displays the regression analyses with control variables alone and with control variables and predictors. For the first analysis, it shows a significant positive association between a high level of education and loneliness, and a significant negative one between social network size and loneliness. For the final regression, the table depicts significant positive associations between education, Interpersonal Sensitivity, Depression, and loneliness, and a significant negative one between Somatization and loneliness.

**Table 2:** Prediction of loneliness by control variables without (1) and with (2) the nine SCL-90 symptom dimensions (standardized regression coefficients; *N* = 397)

	Loneliness	
	1	2
Age	− 0.02	0.06
Gender (“female”)^1^	− 0.03	0.03
Education (“no certification”)		
low	0.06	0.07
moderate	0.12	0.16*
high	0.20**	0.24***
Living with partner (“no”)	− 0.05	− 0.03
Social network size	− 0.16**	− 0.06
Somatization		− 0.21**
Obsessive-compulsive		− 0.08
Interpersonal sensitivity		0.25**
Depression		0.26***
Anxiety		− 0.02
Anger-Hostility		− 0.05
Phobic Anxiety		0.06
Paranoid Ideation		0.10
Psychoticism		0.09
R^2^	0.04	0.24

Note. *p ≤ 0.05; **p ≤ 0.01; ***p ≤ 0.001.

^1^The category coded as “0” (= reference category) is presented in parentheses.

## Discussion

Our results showed significant connections between higher levels of Interpersonal Sensitivity and Depression as well as lower levels of Somatization and loneliness but there were no significant associations between any of the other SCL-90 dimensions and loneliness. To our knowledge, this is the first study to analyze the associations between psychological symptoms (at the level of symptom dimensions) and loneliness in a sample of people who are unemployed and diagnosed with a mental illness. Hence, our results may help to shed light on a highly vulnerable and often neglected group. By identifying those symptom dimensions that are related to loneliness, we can contribute to theory building and intervention development.

Our results showed a higher GSI score in our sample, i.e. a higher symptom level, compared to samples in Germany and the Ukraine, and also compared to German samples of primary care as well as psychosomatic outpatients [29–31]. While the GSI in our sample (1.4) was markedly higher than that reported in the German study with university members (0.3; [29]), it is close to the score found in a German sample of young adults with mental illnesses (1.3; [32]). International studies corroborate our results, showing that unemployment as well as mental illnesses, i.e., affective disorders and substance use disorder, may increase GSI scores [33–35]. In addition, participants in our sample exhibited elevated levels of social isolation, in terms of network size, and loneliness [36, 37], reflecting other research that indicates relationships between unemployment as well as mental illness and loneliness [10].

The results indicated a high level of Somatization, comparable to that observed in patients with affective disorders [35], and a significant negative association between Somatization and loneliness. This is in contrast with the findings of previous studies that showed positive associations between somatization and loneliness [38, 39] and high levels of loneliness in patients diagnosed with somatic symptom disorder [40]. One potential explanation for this discrepancy is that somatization exerts varying effects across different demographic groups. For example, while somatization frequently results in social withdrawal and the subsequent experience of loneliness, these processes may be less relevant in the context of UMIs. I.e., given that UMIs in our sample exhibit high levels of social isolation in general, somatization may also have beneficial effects for them, e.g., when it leads to more social interaction via the utilization of health services [41, 42]. It is also worth noting that while the association between Somatization and loneliness was negative in the regression analysis, our previous correlational analysis showed a small but positive association between the two. Given that our analyses suggest no significant multicollinearity, the results may point to a more intricate relationship and the potential influence of moderating variables that are partially reflected in the control variables included in the regression, e.g., physical health status or personality traits. Further research is required to ascertain the underlying causes and quantify the observed relationships.

Interpersonal Sensitivity refers to feelings of personal inadequacy, self-deprecation, and acute self-consciousness in social interaction [43]. The level of Interpersonal Sensitivity in our sample is almost four times as high as in the German ample of university members and clearly above levels from primary care and psychosomatic patient populations [29]. Our results indicate a significant and positive association between the SCL-90 dimension and loneliness, matching with the literature that shows cross-sectional associations between Interpersonal Sensitivity and loneliness in Chinese gay men [44] and a longitudinal association between rejection sensitivity – a concept close to Interpersonal Sensitivity – and later loneliness [45], a process that could be mediated by mechanism of social withdrawal [46]. On the other hand, a lack of social support had been shown to contribute to higher levels of Interpersonal Sensitivity later on [47]. Given that participants in our sample exhibited low levels of social network size and high levels of loneliness, it is evident that social interaction and social competence should be a fundamental aspect of mental health prevention strategies for unemployed individuals. One potential approach to achieve this could be the prescription of social activities, as proposed by Chatterjee et al. [48]. This approach could be readily integrated into the existing system of support measures for individuals without employment.

Participants in our sample exhibited high levels of Depression, slightly higher that those reported for German psychosomatic outpatients [29], and there was a significant positive association between Depression and loneliness. This finding is consistent with the existing literature, which demonstrates the bidirectional relationship between loneliness and depression [3, 49]. This suggests that both conditions are risk factors for each other and may also reinforce each other. In addition, high levels of depression are associated with a variety of (mental) health problems, like chronic stress, suicidality, and cognitive decline in older age [50–52]. Our results underscore the necessity of diagnosing and treating depressive disorders in the UMI population. Furthermore, an active, outreach-based approach to prevention is indicated, given the frequently observed reluctance of individuals to seek assistance from the healthcare system independently [53]. In addition, digital applications designed to address subclinical levels of depression may prove beneficial in providing accessible support [54, 55].

Our results showed no significant associations between Obsessive-Compulsive, Anxiety, Anger-Hostility, Phobic Anxiety, Paranoid Ideation, Psychoticism, and loneliness. While the relevance of these domains may be limited with regard to loneliness, our descriptive findings indicate that UMIs exhibit a higher burden on all nine SCL-90 dimensions when compared to the general population, as well as to primary care and psychosomatic outpatients (with the exception of anxiety in psychosomatic outpatients; [29]). This is a significant issue, as elevated levels of SCL-90 domain scores may influence UMIs in numerous ways, impacting both wellbeing and (re)integration into the labor market.

We identified a significant positive relationship between our control variable, education, and loneliness. This finding may be due to the fact that individuals with higher levels of education are more likely to also have friends with higher levels of education – a principle often referred to as the law of homophily [56] – who are less likely to experience unemployment [57, 58], making unemployed individuals in highly educated networks feel even more isolated. On the other hand, unemployment may be more common and less stigmatized in the networks of people with lower levels of education, leaving these individuals feeling less lonely.

This study has several notable strengths, including a sample drawn from a population that is critical in terms of public mental health yet dramatically underresearched. However, it also has some limitations, such as the cross-sectional nature of the dataset, which limits the ability to draw causal inferences. Additionally, there may be other potential predictors that could be included in future studies, such as the role of social networking sites and media use in relation to health and loneliness [59]. It would be beneficial for future research to consider these factors and employ a longitudinal design.

## Data Availability

The data that support the findings of this study are available from the corresponding author upon reasonable request.
